# Temporal Heterogeneous in the Effectiveness of Inactivated CoronaVac and Sinopharm Vaccines Against SARS-CoV-2 Reinfections in China

**DOI:** 10.1155/2024/9533861

**Published:** 2024-10-07

**Authors:** Shihong Yang, Hualei Xin, Xingying Lang, Jin Hua, Xiaoman Cui, Lu Li, Chuchu Ye, Ying Qin, Yu Li, Ben Cowling, Shengjie Lai, Ke Sun, Zhongjie Li

**Affiliations:** ^1^ Division of Infectious Disease Dalian Center for Disease Control and Prevention, Dalian, Liaoning Province, China; ^2^ School of Population Medicine and Public Health Chinese Academy of Medical Sciences and Peking Union Medical College, Beijing, China; ^3^ World Health Organization Collaborating Centre for Infectious Disease Epidemiology and Control School of Public Health Li Ka Shing Faculty of Medicine The University of Hong Kong, Hong Kong, China; ^4^ Department of Infectious Disease Control and Prevention Shanghai Pudong New Area Center for Disease Control and Prevention, Shanghai, China; ^5^ Division of Infectious Disease Chinese Center for Disease Control and Prevention, Beijing, China; ^6^ WorldPop School of Geography and Environmental Science University of Southampton, Southampton, UK; ^7^ Institute of Life Science University of Southampton, Southampton, UK

**Keywords:** China, COVID-19, inactivated vaccination, reinfection

## Abstract

We aimed to understand the temporal dynamics of SARS-CoV-2 reinfection risk and assess the impact of inactivated vaccination on the occurrence of reinfection. We investigated the reinfection risk of SARS-CoV-2 from November 1, 2022, to February 12, 2023, when China rapidly lifted the zero-COVID policy. The study subjects were those who were first infected during the zero-COVID period between January 1, 2020, and October 31, 2022, in Dalian city, China. Among the 1961 previous infections, 126 (6.4%, 95% CI: 5.4, 7.5) were reinfected. The risk of reinfection increased over time since initial infection. Compared with those who did not receive or received one dose of inactivated vaccine, receiving two or three doses was associated with additional protection against reinfection among individuals who were infected with pre-Omicron more than a year earlier, with the OR ranged from 0.33 (95% CI: 0.03, 1.83) to 0.91 (95% CI: 0.22, 3.27). In contrast, no protective effect from two or three doses of vaccines against reinfection was observed among those who were first infected with Omicron variants within a year. Primary or booster vaccination contributed to limited protection against reinfection or symptomatic reinfection among individuals infected with Omicron SARS-CoV-2 within a year. However, a booster dose after 1 year of natural infection may provide additional protection against reinfection.

## 1. Introduction

With the ongoing COVID-19 epidemic, a large proportion of people globally have been vaccinated and infected, particularly in the era of the Omicron epidemic [1], which showed marked resistance to neutralization by serum not only from previous infections but also from individuals with primary or booster vaccination [2–6]. People could obtain immunity from natural infection, vaccination, or both. Overall, natural infection confers a higher and more durable protection than vaccination [7–11], and the combined effect of these two forms of immunity, called hybrid immunity, provides higher protection against reinfection compared to the same number of immunity stimulations from natural infection or vaccination alone [7, 12–16].

After almost 3 years of “dynamic zero-COVID” policy, China announced an adjustment of its COVID-19 response strategy on November 1, 2022, in which the quarantine period for inbound travelers and close contacts was shortened, contact tracing was suspended, and the polymerase chain reaction (PCR) testing coverage was restricted, and, further, the relaxation measures for lifting the policy were announced on December 7, 2022, including prohibiting mass testing in the community, and allowed household isolation and quarantine rather than in specialized facilities [17]. Since then, the incidence of Omicron BF.7 and BA.5.2 subvariants of SARS-CoV-2 in China increased rapidly from the start of December 2022, peaked around the end of December, and then declined until the end of January 2023 [17, 18]. One transmission dynamic study based on social media data suggested that over 90% of the population was infected in Beijing, China, between November 1, 2022, and January 31, 2023 [17].

This study explored the temporal dynamics of the reinfection or symptomatic reinfection risk among previously infected individuals by time since the first infection and the protective effect of inactivated vaccines against reinfection or symptomatic reinfection by time since the vaccination.

## 2. Materials and Methods

### 2.1. Data Collection

During the “Dynamic zero-COVID” period in China before November 1, 2022, almost all laboratory-confirmed COVID-19 cases by RT-PCR were admitted to the hospital and reported to the national infectious diseases surveillance systems, and a detailed epidemiological investigation was conducted for each patient [19]. In this study, individual information on basic demographic (sex, age, occupation, and residential address), symptom onset dates, and disease severity (asymptomatic, mild, moderate, severe, and critical; Supporting Information) for laboratory-confirmed COVID-19 cases diagnosed before November 1, 2022, in Dalian city, China, was extracted from the national infectious diseases surveillance system. To identify the impact of virus strains on the occurrence of SARS-CoV-2 reinfection, we collected information on the infected virus strains (ancestral strain, Delta, Omicron BA.1, BA.2, and BA.5) for each case during their first infection from the individual investigation reports, given sequenced genome testing was conducted for index cases in all local SARS-CoV-2 outbreaks and also for some sporadic cases. Vaccination might play an essential role in preventing reinfection. Thus, individual vaccination status, including brand, type, doses received, and dates for each dose, was collected from the National Integrated Information Platform for immunization programs. Cases were defined as receiving incomplete vaccination series, primary vaccination series, and booster vaccination series if they had received one, two, or three doses of vaccines 14 days before December 22, 2022, and the epidemic peak of SARS-CoV-2 outbreak occurred after abandoning the “Dynamic zero-COVID” policy since November 1, 2022 [17–19]. The primary and booster series of vaccines were further categorized as within 6 months, 6–12 months, or over 12 months if the last dose was received within 6 months, 6–12 months, or over 12 months, respectively, 14 days before December 22, 2022. People who had an invalid vaccination date had received a noninactivated vaccine or had received a fourth dose before reopening was excluded. We also excluded sporadic cases since most were imported cases, their vaccination status was incomplete in the National Immunization Information System (some of them might receive vaccination outside China).Cases younger than or equal to 3 years old and those with reinfection documented during the “Dynamic zero-COVID” period were also exclueded.

We then conducted a telephone survey by trained interviewers between February 12 and 17, 2023 to ascertain the reinfection status among the above-identified previous infections during the reopening period after November 1, 2022, when a large epidemic of infections began. Reinfection was defined as between November 1, 2022, and February 12, 2023, previous infections with positive PCR or rapid antigen test (RAT) results, or previous infections without laboratory testing but had at least one SARS-CoV-2-related symptom, such as fever, cough, weakness, dizziness and headache, myalgia, pharyngula, and expectoration, and with at least one laboratory-confirmed case in their family. We defined symptomatic reinfection as the presence of SARS-CoV-2-related symptoms with or without laboratory testing. Reinfection or symptomatic reinfection was only confirmed for those with a reinfection date at least 90 days from the symptom onset date of the first infection [1]. Other information, such as medical or personal hygiene behaviors during the reopening period, was also collected in the telephone survey. Individuals aged <12 years were required to finish the survey by their parents or legal guardians.

### 2.2. Statistical Analysis

Overall and subgroup-specific risks of reinfection or symptomatic reinfection were estimated as the proportion of reinfections among previous infections. Time since the first infection for each case was calculated as the interval between the symptom onset date during the first infection and the referent date of December 22, 2022, the epidemic peak of SARS-CoV-2 after reopening [17, 18]. To find the temporal changes of reinfection or symptomatic reinfection risk with time since the first infection, multivariate logistic regression was conducted using reinfection, symptomatic reinfection, or not as dependent variable; time since the first infection as the independent variable; controlling sex, age, underlying conditions, and disease severity during the first infection (asymptomatic, mild, moderate, severe, or critical), the virus strains infected during the first infection (pre-Omicron and Omicron), and vaccination status (zero or one dose, two doses within 6 months, two doses between 6 and 12 months, two doses over 12 months, three doses within 6 months, three doses between 6 and 12 months, and three doses over 12 months). Then, we extracted the adjusted probability of reinfection or symptomatic reinfection for each individual from the logistic regression results and a point plot with means and 95% CIs of the adjusted probability of reinfection or symptomatic reinfection with periods of 3–6 months, 6–12 months, 12–18 months, and 18–36 months after the first infection was created to indicate the temporal dynamic of the reinfection risk.

To evaluate the effectiveness of inactivated vaccination on reinfection or symptomatic reinfection among previous infections, we first stratified the data by time since the first infection as over 12 months with pre-Omicron infection and within 12 months with Omicron infection. For each dataset, we conducted multivariate logistic regressions using reinfection or symptomatic reinfection as the dependent variable; vaccination status (same categorization as the above analysis, zero or one dose as the referent group) as the independent variable; and controlling sex, age, underlying conditions, disease severity during first infection, and time from the first infection. Results were expressed as the adjusted Odds Ratio (aOR) in each vaccination group relative to the referent groups.

Nonparametric bootstrap approach with 1000 resamples was used to obtain the uncertainties of the overall risk of reinfection or symptomatic reinfection. R version 4.1.0 was used to carry out all analyses.

## 3. Results

### 3.1. Study Subjects

Among the 2872 participants who were first infected during the “Dynamic zero-COVID” period between January 1, 2020, and October 31, 2022, in Dalian City, China, we excluded 187 nonpermanent residents, 575 who could not be contacted or refused to participate, 96 sporadic cases, 40 younger or equal to 3 years old, 12 who had received one or more doses of noninactivated vaccines, and one who had a reinfection documented during the “Dynamic zero-COVID” period, leaving a cohort of 1961 (68.3%, 1961/2872) participants in the final analysis (Figure S1). The enrolled individuals showed an older age and a shorter time since the first infection than the overall 2872 previous infections (Table S1).

**Figure 1:**
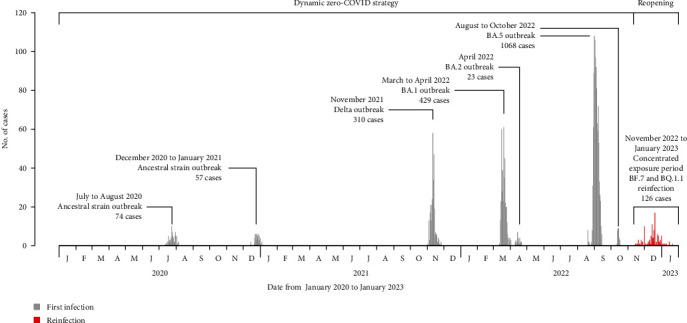
Epidemic curve of the first-time infections and reinfections from January 1, 2020, to February 12, 2023, in Dalian city, China.

Among the cohort of 1961 individuals, 48.6% were males; the median age was 44.0 years (IQR: 31.0, 57.0), with 75.6% aged between 15 and 64 years. Most (74.5%) presented no symptoms during their first infection. The 1961 participants were from seven local outbreaks, including two outbreaks in 2020 caused by ancestral strain, one Delta outbreak in November 2021, one BA.1 and one BA.2 outbreak in early 2022, and two BA.5 outbreaks in late 2022. The proportions of participants with time since the first infection of within 6 months, between 6 and 12 months, between 12 and 18 months, and over 18 months were 54.5%, 23.0%, 15.8%, and 6.7%, respectively, which were corresponding to the proportions of infections with BA.5, BA.1 or BA.2, Delta, and ancestral strain. Most participants (1670, 85.2%) had received primary or booster vaccines; 20 (1.0%), 145 (7.4%), and 287 (14.6%) received primary vaccines within 6 months, between 6 and 12 months, and over 12 months, respectively; and 45 (2.3%), 863 (44.0%), and 310 (15.8%) received booster vaccines within 6 months, between 6 and 12 months, and over 12 months, respectively (Table 1 and Figure 1).

### 3.2. Risk of Reinfection and Symptomatic Reinfection

**Figure 2:**
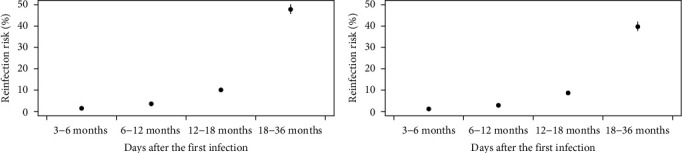
Risk of reinfection (A) and symptomatic reinfection (B) during the reopening period between November 1, 2022, and February 12, 2023 with time since the first infection among those who were first infected during the “Dynamic-zero COVID” period between January 1, 2020 and October 31, 2022 in Dalian city, China.

Among the 1961 previous infections, 126 (6.4%, 95% CI: 5.4, 7.5) were reinfected, and 105 (5.4%, 95% CI: 4.3, 6.4) had symptomatic reinfections (Table 1). Among the 126 reinfections, 21 (16.7%) were confirmed by PCR or RAT without symptoms, 46 (36.5%) were confirmed by PCR or RCT and presenting symptoms, and 59 (46.8%) were confirmed by SARS-CoV-2-related symptoms alone. Only 2 (1.6%, 2/126) required admission to hospital. Similar to the overall epidemic curve of COVID-19 after reopening in China, the number of reinfections identified in this study increased from mid-November 2022, peaked around December 22, 2022, and then declined until the end of January 2023 (Figure 1). By fitting a regression model, we found an increasing trend of reinfection and symptomatic reinfection risk with time since the first infection (Figure 2 and Table S2). The adjusted mean risk of reinfection and symptomatic reinfection was 1.5% (95% CI: 1.4, 1.6) and 1.2% (95% CI: 1.1, 1.3) 3–6 months after the first infection, 3.6% (95% CI: 3.5, 3.8) and 2.9% (95% CI: 2.8, 3.0) 6–12 months after the first infection, 10.1% (95% CI: 9.6, 10.7) and 8.7% (95% CI: 8.2, 9.2) 12–18 months after the first infection, and 47.8% (95% CI: 45.7, 50.2) and 39.7% (95% CI: 37.7, 42.1) 18–36 months after the first infection (Figure 2).

### 3.3. Effectiveness of Inactivated Vaccine Against Reinfection or Symptomatic Reinfection

We further evaluated the effectiveness of inactivated vaccines against reinfection or symptomatic reinfection with time since the first infection. For previous Omicron BA.1, BA.2, and BA.5 infections within 12 months, the aORs of getting reinfection or symptomatic reinfection among those who had completed primary or booster series of vaccination with different times of last dose were generally over 1, using previous infections without vaccination or with one dose of vaccine as the referent group. In contrast, the aORs were lower than 1 for previous ancestral strain or Delta infections over 12 months, with statistically significant protection against reinfection identified among those who had received booster series of vaccines between 6 and 12 months (aOR: 0.44, 95% CI: 0.22, 0.85). A booster vaccination within 6 months before exposure presented the lowest risk of symptomatic reinfection among those who were previously infected over 12 months (aOR: 0.33, 95% CI: 0.03, 1.83; Table 2).

## 4. Discussion

By investigating the risk of reinfection and symptomatic reinfection during a concentrated exposure period among those who were first infected during the “Dynamic zero-COVID” in China, we found that the risk of reinfection and the risk of symptomatic reinfection was 6.4% and 5.4%, respectively, with an increasing trend with time since the first infection. Inactivated vaccines of Sinopharm and CoronaVac with primary or booster series with different times of last dose provided limited protection against reinfection or symptomatic reinfection for those who were first infected with Omicron within a year. However, for those who had an infection history over a year, primary or booster series of vaccination provided additional protection against reinfection or symptomatic reinfection, compared to previous infections without or with one dose of vaccination, and a booster dose within 6 months before virus exposure showed the highest protection against reinfection.

We observed an increasing trend of reinfection risk over the time since the previous infection, indicating the immune-waning trend of protection from natural infection. We observed that the reinfection risk was about 3.6%–10.1% 12 months after the previous infection, significantly lower than those without a natural infection [17]. Although some asymptomatic cases would be missed in our study, we could still see significant protection from natural infection against reinfection. Similar to our research, one study conducted among adolescents identified around 50% of protection from previous Delta or Omicron infection against Omicron reinfection [13], and another study analyzed the relationship between previous pre-Omicron infection and Omicron BA.1 or BA.2 reinfection and revealed that previous pre-Omicron infection about a year still provided 50.2% of protection against reinfection [9]. However, we also noticed a study that suggested no protection from previous pre-Omicron infection with or without vaccines against symptomatic Omicron reinfection [14]. The persistent protection from previous infection extending to a long time could be due to a broad antibody response recognizing epitopes across multiple viral proteins and other nonstructural proteins less susceptible to mutations [12, 20]. However, due to the overlapping between circulation of SARS-CoV-2 variants and time since the first infection, we should be warned that it was challenging in the data interpretation in this study.

To eliminate the overlapping effect of virus strains and time since the previous infection, we further analyzed the protective effect of inactivated vaccines against reinfection by stratifying the data based on time since the first infection, which also meant stratifying by virus strain (pre-Omicron and Omicron). We found that primary or booster series of inactivated vaccines with different times of the last dose conferred basically no statistical protection against reinfection, compared to natural infections without or with one dose of vaccine. This was similar to another study that proved one vaccine dose among adolescents and convalescent children could not provide enough effect to cope BA.4 or BA.5 reinfection [16]. Previous studies showed that vaccination could provide additive protection against reinfection, symptomatic reinfection, or severe disease among previous infections [9, 11–16, 21]. However, the protection still mainly originated from previous infections. For example, Carazo et al. estimated that previous BA.1 infection was in connection with a 72% reduction of BA.2 reinfection, and the protection was increased to 96% after receiving the primary series of vaccines [12]. Another study identified a similar protective effect against BA.2 reinfection between previous infection alone (52.2%) and previous infection with primary vaccine series (52.0%) [9]. With previous infections without or with incomplete series of vaccines as the referent group in this study, the nonstatistically significant effect of protection from primary or booster series of vaccines could be explained.

Unlike the odds ratios of the effectiveness of primary or booster vaccines against reinfection for those who were first infected within a year, the odds ratios were lower than one among those who were first infected over a year and even had statistically significant protection against reinfection among those who had completed booster series of vaccination between 6 and 12 months. A similar phenomenon was observed in another hybrid immunity study which identified higher vaccine protection against reinfection among previous infections over a year compared to those up to a year [16]. The waning of naturally acquired immunity might explain this. For previous infections within a year, the immunity from natural infection might be enough to protect against reinfection or symptomatic reinfection; primary or booster series of vaccines might provide limited protection or even increase the risk of reinfection, which was shown in this study (Table 2), possibly attributed to changes in social distancing behaviors after being vaccinated [19]. Surprisingly, we identified a significant increase in reinfection risk (OR: 36.05) among those previously infected with Omicron within a year and received two inactivated vaccines within 6 months before exposure. Although the risk might be overestimated due to the small sample size, that further proved the effectiveness of natural infection and the limited or even reverse impact of inactivated vaccines against reinfection for those with first infection history within a year. For previous infections over 12 months, immunity from naturally acquired waned, and, therefore, we could expect the vaccination benefit to be greater in this group.

For those who had been infected by SARS-CoV-2, they might want to know if an additional vaccine dose after infection could reduce the chance of getting reinfection. One study analyzed the association between one dose of mRNA vaccine after infection from previous and the reinfection occurrence and found significant protection against Delta, BA.1, and BA.2 but no protection effect for BA.4 and BA.5 [16]. Our study implied some additional protection from a booster dose of the inactivated vaccine among previous infections over a year compared to those who had already received two doses, and the protection was more obvious against symptomatic reinfection among those who had received a third dose within 6 months before virus exposure. This result was consistent with previous studies that a booster dose among previous infections provided only a short period of additional protection against reinfection compared to previous infections with two doses of vaccines [9].

Our study has limitations. First, as we mentioned, the information on virus strains and the time since the first infection highly overlapped. Therefore, interpreting the result of reinfection or symptomatic risk with time since the first infection would be challenging as we couldn't include the full effects of virus strains in the model. To overcome this, we stratified the data by time from the first infection as pre-Omicron period and Omicron period and fitted the model in each dataset; however, the smaller sample size after stratification could possibly lead to some inaccuracy of some estimates. In addition, based on the definition of reinfection used in this study, we might underestimate the reinfection risk given the unidentified asymptomatic infections, which are less likely to be tested.

## 5. Conclusions

With more and more people having already been infected with SARS-CoV-2 and receiving primary or booster series of vaccines, our findings have important implications for preparedness and response to future epidemic waves. An increasing trend of reinfection or symptomatic reinfection risk of SARS-CoV-2 with time since the first infection was observed, with a low risk identified within a year. Natural infection within a year provided sufficient protection against reinfection, and primary and booster series of inactivated vaccination provided more benefit among those who had an infection history over a year. In addition, a booster dose after 1 year of natural infection could be recommended.

## Figures and Tables

**Table 1:** Basic characteristics of the enrolled participants and the risk of infection and symptomatic reinfection.

Characteristics	No. of enrolled (% of column)	No. of reinfections (% of row)	No. of symptomatic reinfections (% of row)
Overall	1961 (100.0)	126 (6.4)	105 (5.4)
Sex			
Male	954 (48.6)	53 (5.6)	43 (4.5)
Female	1007 (51.4)	73 (7.2)	62 (6.2)
Age, years, and median (median, IQR^a^)	44.0 (31.0, 57.0)		
4-	204 (10.4)	12 (5.9)	10 (4.9)
15-	790 (40.3)	66 (8.4)	57 (7.2)
45-	693 (35.3)	39 (5.6)	32 (4.6)
65-	274 (14.0)	9 (3.3)	6 (2.2)
Severity at first infection			
Asymptomatic	1461 (74.5)	51 (3.5)	44 (3.0)
Mild	253 (12.9)	23 (9.1)	20 (7.9)
Moderate	235 (12.0)	48 (20.4)	37 (15.7)
Severe or critical	12 (0.6)	4 (33.3)	4 (33.3)
Underlying conditions			
Yes	281 (14.3)	17 (6.0)	14 (5.0)
No	1680 (85.7)	109 (6.5)	91 (5.4)
Variants at first infection			
Ancestral strain	131 (6.7)	60 (45.8)	48 (36.6)
Delta	310 (15.8)	34 (11.0)	31 (10.0)
BA.1	429 (21.9)	25 (5.8)	21 (4.9)
BA.2	23 (1.2)	1 (4.3)	1 (4.3)
BA.5	1068 (54.5)	6 (0.6)	4 (0.4)
COVID-19 vaccine doses			
0 dose	224 (11.4)	22 (9.8)	17 (7.6)
1 dose	67 (3.4)	12 (17.9)	9 (13.4)
2 doses within 6 months^b^	20 (1.0)	3 (15.0)	1 (5.0)
2 doses within 6–12 months^c^	145 (7.4)	7 (4.8)	7 (4.8)
2 doses over 12 months^d^	287 (14.6)	16 (5.6)	13 (4.5)
3 doses within 6 months^b^	45 (2.3)	3 (6.7)	2 (4.4)
3 doses within 6–12 months^c^	863 (44.0)	46 (5.3)	38 (4.4)
3 doses over 12 months^d^	310 (15.8)	17 (5.5)	18 (5.8)
Time since the first infection month, median, IQR)	3.8 (3.6, 9.4)		
Within 6 months	1068 (54.5)	6 (0.6)	4 (0.4)
Between 6 and 12 months	452 (23.0)	26 (5.8)	22 (4.9)
Between 12 and 18 months	310 (15.8)	34 (11.0)	31 (10.0)
Over 18 months	131 (6.7)	60 (45.8)	48 (36.6)

^a^IQR, interquartile range.

^b^Received the last dose of vaccination within 6 months before December 22, 2022.

^c^Received the last dose of vaccination between 6 and 12 months before December 22, 2022.

^d^Received the last dose of vaccination over 12 months before December 22, 2022.

**Table 2:** Effectiveness of inactivated vaccines on the occurrence of overall reinfection and symptomatic reinfection among previous infections based on logistic regression, stratified analysis by time since the first infection.

Characteristics	No. of reinfections (%)	No. of symptomatic reinfections (%)	Adjusted OR for reinfection (95% CI^a^)	Adjusted OR for symptomatic reinfection (95% CI^a^)
Previous pre-Omicron infection over 12 months				
COVID-19 vaccine doses				
0 or 1 dose (*N* = 95)	32 (33.7)	25 (26.3)	Referent	Referent
2 doses within 6 months^b^ (*N* = 5)	1 (20.0)	0 (0.0)	0.77 (0.04–6.46)	NA
2 doses between 6 and 12 months^c^ (*N* = 14)	4 (28.6)	4 (28.6)	0.59 (0.14–2.14)	0.91 (0.22–3.27)
2 doses over 12 months^d^ (*N* = 69)	10 (14.5)	8 (11.6)	0.47 (0.19–1.10)	0.54 (0.20–1.32)
3 doses within 6 months^b^ (*N* = 19)	3 (15.8)	2 (10.5)	0.46 (0.08–1.99)	0.33 (0.03–1.83)
3 doses between 6 and 12 months^c^ (*N* = 147)	30 (20.4)	26 (17.7)	0.44 (0.22–0.85)	0.57 (0.28–1.14)
3 doses over 12 months^d^ (*N* = 92)	14 (15.2)	14 (15.2)	0.58 (0.25–1.30)	0.86 (0.37–1.97)
Previous Omicron infection within 12 months				
COVID-19 vaccine doses				
0 or 1 dose (*N* = 196)	2 (1.0)	1 (0.5)	Referent	Referent
2 doses within 6 months (*N* = 15)	2 (13.3)	1 (6.7)	36.05 (3.22–427.43)	26.36 (0.87–807.77)
2 doses between 6 and 12 months (*N* = 131)	3 (2.3)	3 (2.3)	0.82 (0.12–7.03)	2.19 (0.23–48.89)
2 doses over 12 months (*N* = 218)	6 (2.8)	5 (2.3)	3.29 (0.70–23.64)	6.61 (0.97–132.15)
3 doses within 6 months (*N* = 26)	0 (0.0)	0 (0.0)	—	—
3 doses between 6 and 12 months (*N* = 716)	16 (2.2)	12 (1.7)	1.82 (0.48–12.00)	2.47 (0.45–46.02)
3 doses over 12 months (*N* = 218)	3 (1.4)	4 (1.8)	2.15 (0.34–17.17)	6.14 (0.85–124.14)

^a^Adjusting for age, sex, severity at the previous infection, underlying conditions, and time from the first infection.

^b^Received the last dose of vaccination within 6 months before December 22, 2022.

^c^Received the last dose of vaccination between 6 and 12 months before December 22, 2022.

^d^Received the last dose of vaccination over 12 months before December 22, 2022.

## Data Availability

The data that support the findings of this study are available from the corresponding author upon reasonable request.

## Supporting Information

Supporting Information Table S1: characteristic differences between all infections and enrolled subjects. Table S2: impact of time since the first infection on reinfection. Figure S1: flowchart of subject selection.
