# Supplementary material for: Temporal Heterogeneous in the Effectiveness of Inactivated CoronaVac and Sinopharm Vaccines Against SARS-CoV-2 Reinfections in China
Source: Transbound Emerg Dis. 2024 Oct 7;2024:9533861. doi: 10.1155/2024/9533861 (PMC12017158; doi:10.1155/2024/9533861)
Supplement: Supporting Information — Table S1: characteristic differences between all infections and enrolled subjects. Table S2: impact of time since the first infection on reinfection. Figure S1: flowchart of subject selection. [file 9533861.f1.docx]

Temporal heterogeneous in the effectiveness of inactivated CoronaVac and Sinopharm vaccines against SARS-CoV-2 reinfections in China

Supplementary materials

Definition of severity at first infection

The clinical severity used in this study follows the Guidelines in Diagnosis and Treatment of COVID-19 (8th version) published by the National Health Commission in China on 15 April 2021. Asymptomatic SARS-CoV-2 infections were defined as those without presenting clinical symptoms but had positive results from PCR or RAT testing. Mild type is with mild clinical symptoms and without lung infections indicated by a chest X-ray examination. Moderate type means a fever, respiratory tract symptoms, with lung infections indicated by a chest X-ray examination. A person was defined as severe if the patient showed one of the criteria: (1) shortness of breath; (2) Oxygen saturation ≤93% when inhaling air at rest condition; (3) PaO2/FiO2 <=300mmHg (1mmHg=0.133kPa); (4) The clinical symptoms were progressively aggravated, and the lung imaging showed significant lesion progression >50% within 24 ~ 48 hours. A person was defined as critical if the patient showed one of the criteria: (1) Respiratory failure and mechanical ventilation are required; (2) Shock; (3) Combined other organ failure and ICU care is required.

Table S1. Characteristic differences between all infections before November 1, 2022, in Dalian city, China, and enrolled subjects in this study.

| Characteristics | No. all first infections  (% of column) | No. enrolled (% of column) | *P* value from  chi square test |
| --- | --- | --- | --- |
| Overall | 2872 (100.0) | 1961 (100.0) |  |
| Sex |  |  |  |
| Male | 1449 (50.5) | 954 (48.6) | 0.23 |
| Female | 1423 (49.5) | 1007 (51.4) |  |
| Age, years, median (Median, IQR) |  |  |  |
| 0- | 298 (10.4) | 204 (10.4) | <0.01 |
| 15- | 1302 (45.3) | 790 (40.3) |  |
| 45- | 938 (32.7) | 693 (35.3) |  |
| 65- | 334 (11.6) | 274 (14.0) |  |
| Severity at first infection |  |  |  |
| Asymptomatic | 2138 (74.4) | 1461 (74.5) | 0.79 |
| Mild | 350 (12.2) | 253 (12.9) |  |
| Moderate | 364 (12.7) | 235 (12.0) |  |
| Severe or critical | 20 (0.7) | 12 (0.6) |  |
| Time since the first infection (Month) |  |  |  |
| Within 6 months | 1291 (45.0) | 1068 (54.5) | <0.01 |
| Between 6-12 months | 950 (33.1) | 452 (23.0) |  |
| Between 12-18 months | 389 (13.5) | 310 (15.8) |  |
| Over 18 months | 242 (8.4) | 131 (6.7) |  |

Table S2. Impact of time since the first infection on the occurrence of overall reinfection and symptomatic reinfection based on logistic regression.

| Characteristics | Adjusted OR for reinfection (95% CI) ^a^ | Adjusted OR for symptomatic reinfection (95% CI) ^a^ |
| --- | --- | --- |
| Sex |  |  |
| Male | Referent | Referent |
| Female | 1.51 (0.98-2.32) | 1.60 (1.02-2.54) |
| Age | 0.99 (0.98-1.01) | 0.99 (0.98-1.01) |
| Severity at first infection |  |  |
| Asymptomatic | Referent | Referent |
| Mild | 1.23 (0.65-2.28) | 1.13 (0.57-2.17) |
| Moderate | 1.02 (0.55-1.90) | 0.81 (0.42-1.56) |
| Severe or critical | 5.24 (0.96-24.70) | 6.98 (1.25-35.00) |
| Underlying conditions |  |  |
| Yes | 0.98 (0.50-1.86) | 1.04 (0.50-2.04) |
| No | Referent | Referent |
| Variants |  |  |
| Pre-Omicron | Referent | Referent |
| Omicron | 0.81 (0.36-1.83) | 0.65 (0.27-1.56) |
| COVID-19 Vaccine doses |  |  |
| 0 or 1 dose | Referent | Referent |
| 2 doses within 6 months ^b^ | 3.18 (0.61-12.65) | 0.98 (0.05-6.35) |
| 2 doses between 6-12 months ^c^ | 0.56 (0.20-1.42) | 0.83 (0.29-2.17) |
| 2 doses over 12 months ^d^ | 0.76 (0.37-1.54) | 0.86 (0.39-1.85) |
| 3 doses within 6 months ^b^ | 0.52 (0.09-2.06) | 0.36 (0.04-1.92) |
| 3 doses between 6-12 months ^c^ | 0.62 (0.36-1.10) | 0.73 (0.40-1.36) |
| 3 doses over 12 months ^d^ | 0.77 (0.37-1.56) | 1.18 (0.56-2.47) |
| Time since the first infection | 1.01 (1.00-1.01) | 1.01 (1.00-1.01) |

^a^ Adjusting for age, sex, severity at the previous infection, underlying conditions, variants infected during the first infection (pre-Omicron or Omicron) and time from the previous infection.

^b^ Received the last dose of vaccination within six months before December 22, 2022.

^c^ Received the last dose of vaccination between 6-12 months before December 22, 2022.

^d^ Received the last dose of vaccination over 12 months before December 22, 2022.


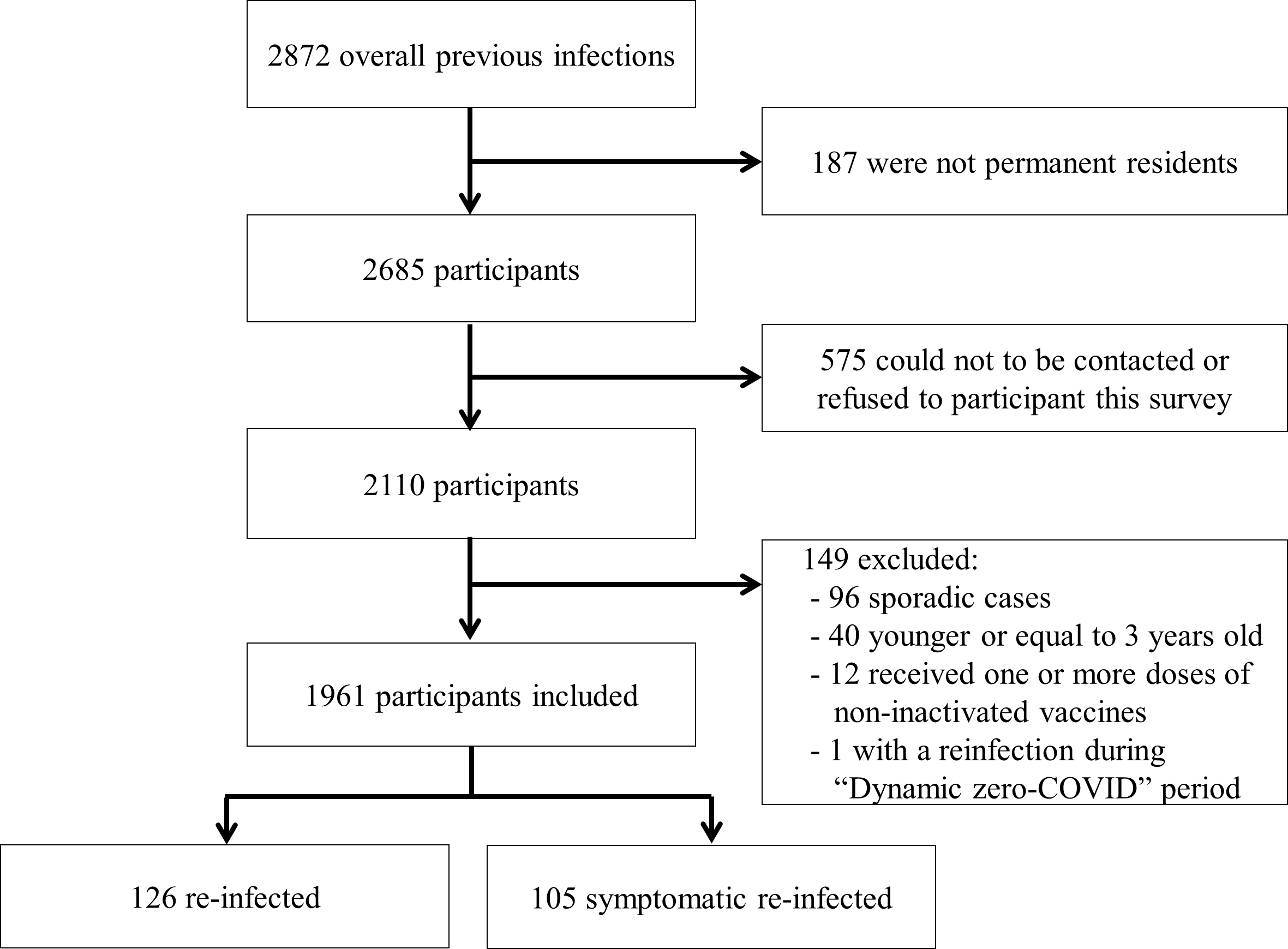


Figure S1. Flowchart of subject selection.
